# Supplementary material for: Comparative Analysis of Circadian Transcriptomes Reveals Circadian Characteristics between Arabidopsis and Soybean
Source: Plants (Basel). 2023 Sep 22;12(19):3344. doi: 10.3390/plants12193344 (PMC10574400; doi:10.3390/plants12193344)
Supplement: Supplementary file 1 [file plants-12-03344-s001.zip › Supplementary figures_XingweiWang et al.pdf]

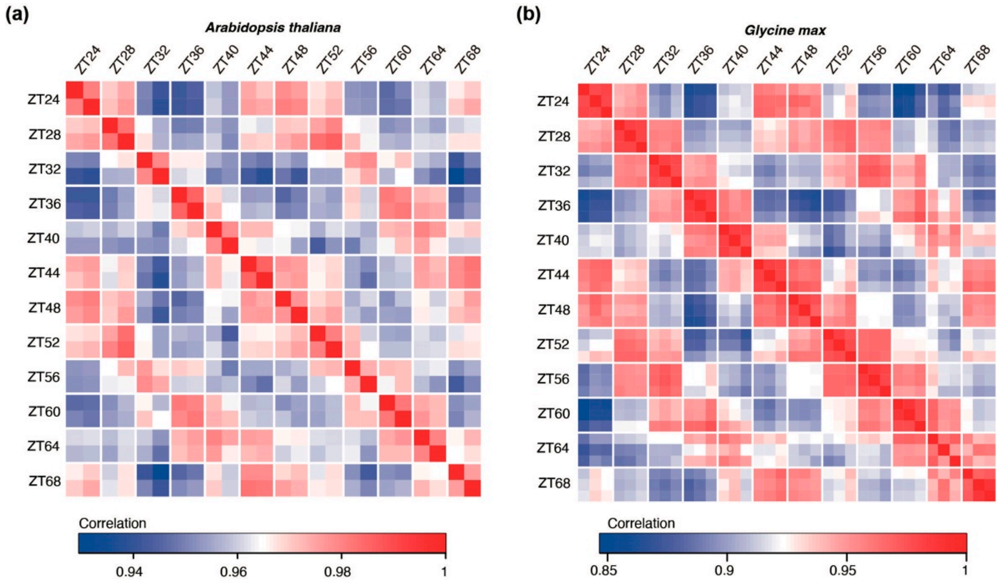

Figure S1. Correlation matrix showing the reproducibility of samples in *Arabidopsis* **(a)** and soybean **(b)** respectively. For each ZT time point, there are 2 replicates for *Arabidopsis* and 3 replicates in soybean.

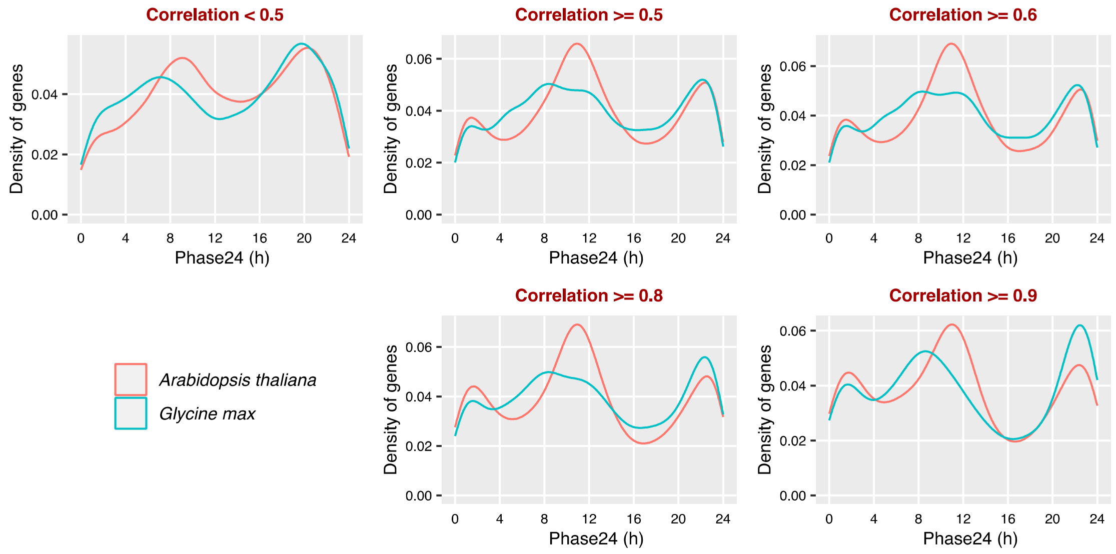

Figure S2. Distribution of phase24 of expressed genes in the harvested samples under the experimental condition in *Arabidopsis* and soybean respectively with correlation < 0.5, correlation >= 0.5, correlation >= 0.6, correlation >= 0.8 or correlation >= 0.9. For all the 5 conditions, *Arabidopsis* significantly differs from soybean with p value < 0.001 (Kolmogorov-Smirnov test). h, hour.

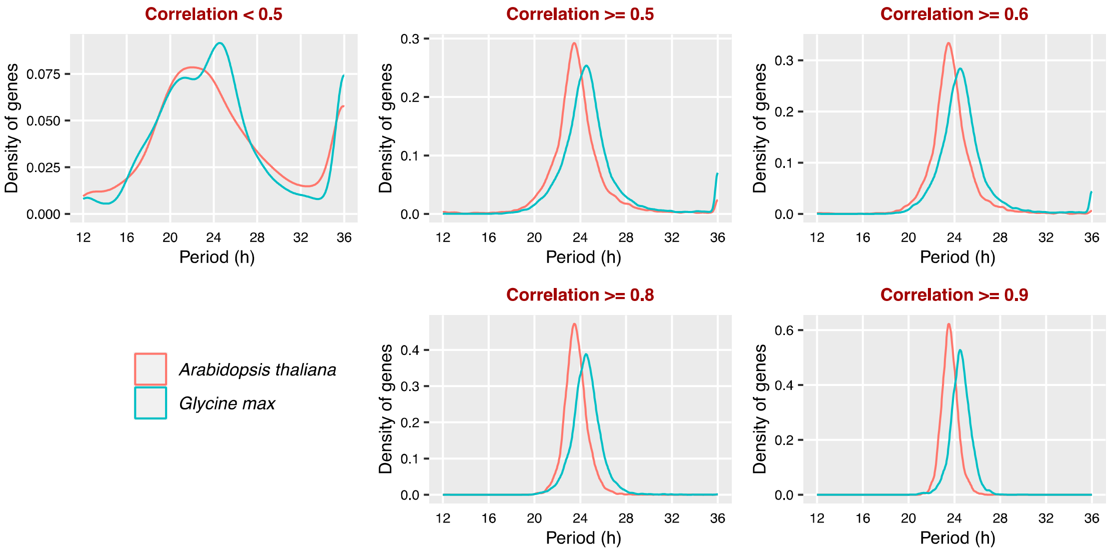

Figure S3. Distribution of period of expressed genes in the harvested samples under the experimental condition in *Arabidopsis* and soybean respectively with correlation < 0.5, correlation >= 0.5, correlation >= 0.6, correlation >= 0.8 or correlation >= 0.9. For all the 5 conditions, *Arabidopsis* significantly differs from soybean with p value < 0.001 (Mann-Whitney test). h, hour.

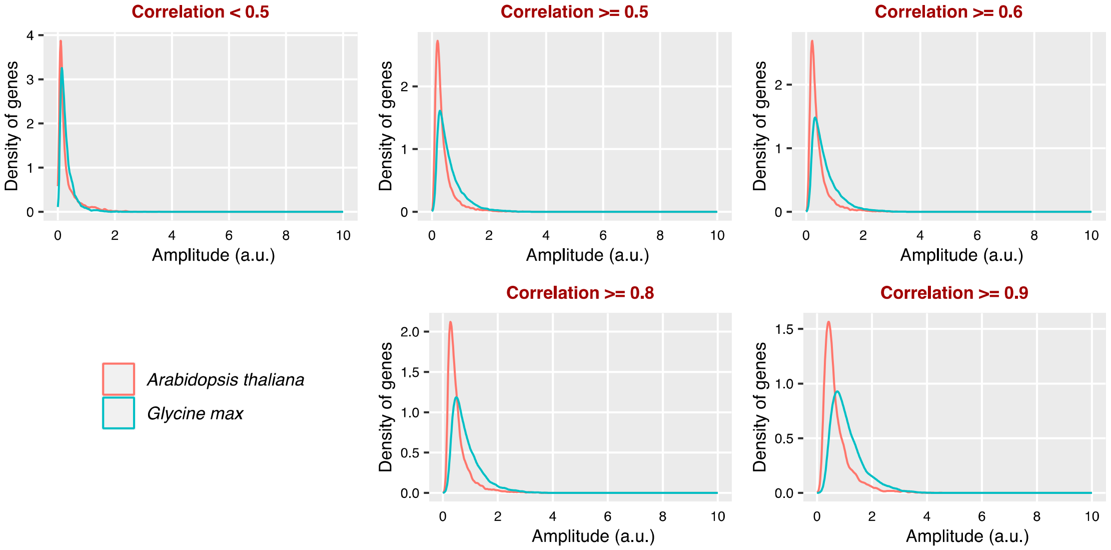

Figure S4. Distribution of amplitude of expressed genes in the harvested samples under the experimental condition in *Arabidopsis* and soybean respectively with correlation < 0.5, correlation >= 0.5, correlation >= 0.6, correlation >= 0.8 or correlation >= 0.9. For all the 5 conditions, *Arabidopsis* significantly differs from soybean with p value < 0.001 (Mann-Whitney test). a.u., arbitrary unit.

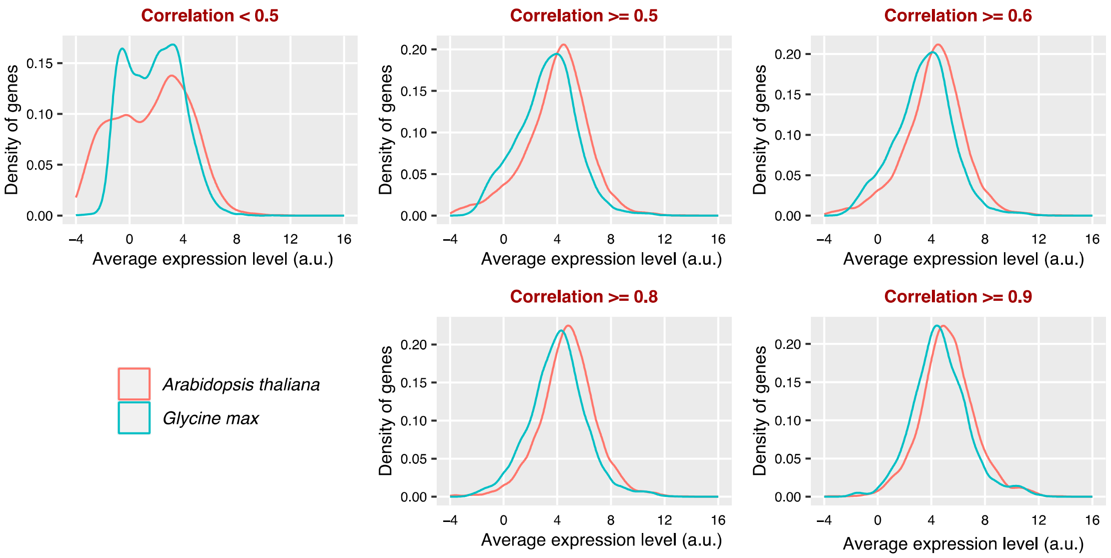

Figure S5. Distribution of average expression level of expressed genes in the harvested samples under the experimental condition in *Arabidopsis* and soybean respectively with correlation < 0.5, correlation >= 0.5, correlation >= 0.6, correlation >= 0.8 or correlation >= 0.9. For all the 5 conditions, *Arabidopsis* significantly differs from soybean with p value < 0.001 (Mann-Whitney test). a.u., arbitrary unit.

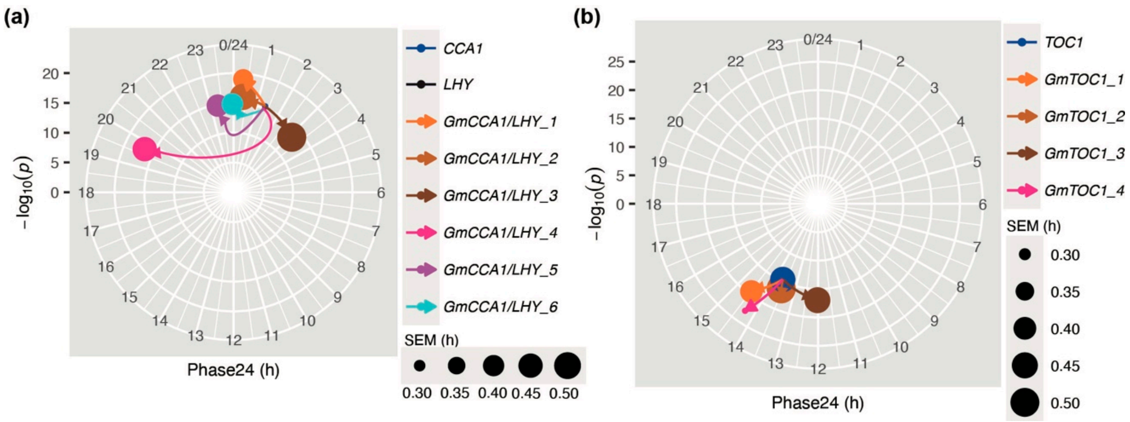

Figure S6. Radial plot showing the differences in phase24 and oscillation robustness between *Arabidopsis CCA1/LHY* (**a**), *TOC1* (**b**) and their homologous genes in soybean. Phase24 indicates gene's phase normalized into a period of 24 h and is plotted as the angular coordinate. Robustness is indicated by $-\log_{10}(p)$ with a larger $-\log_{10}(p)$ representing better oscillation. SEMs are indicated by the size of the symbols. h, hour.

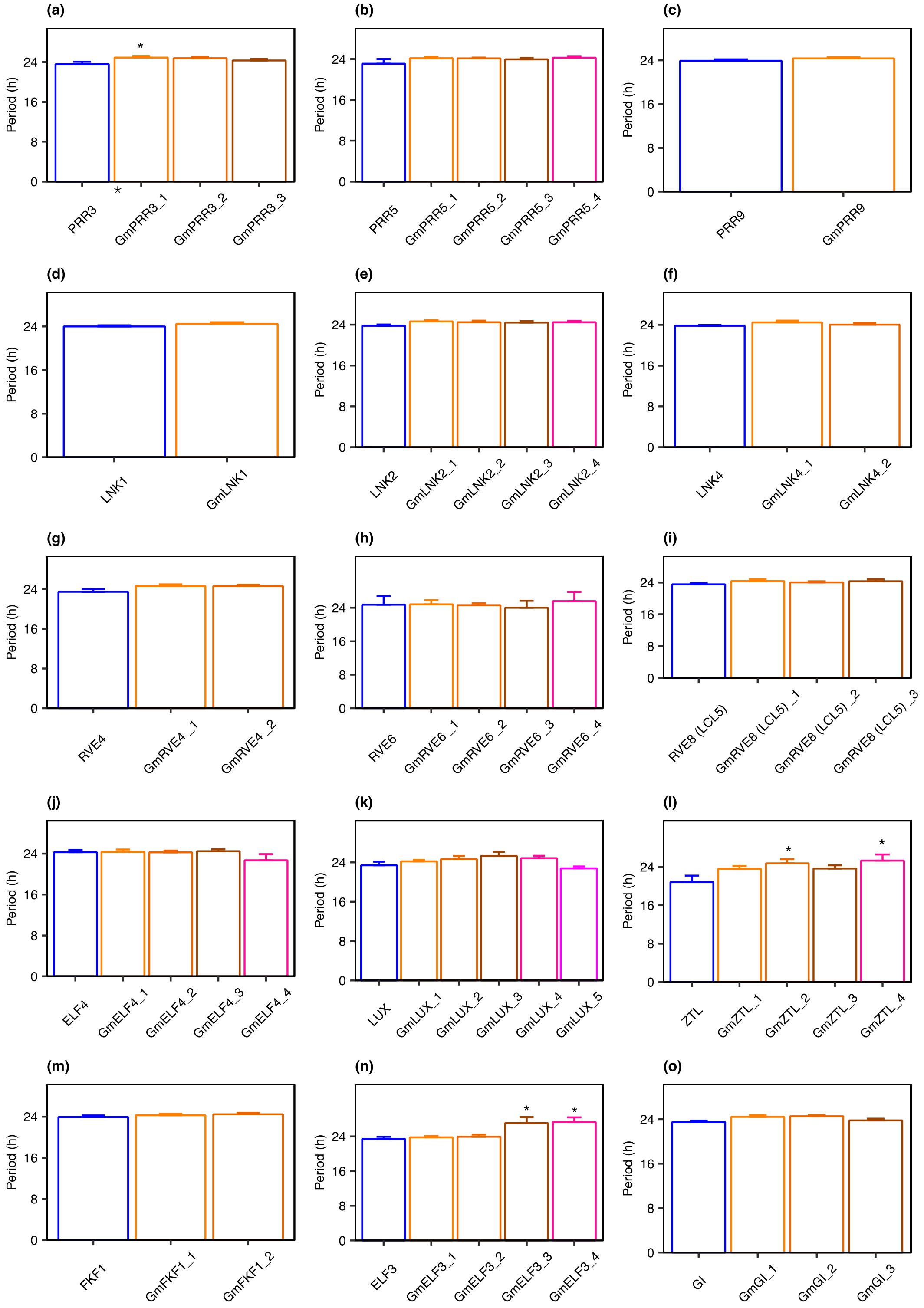

Figure S7. Period of *Arabidopsis* clock genes and their homologous genes in soybean. For bar graph, data were presented as mean + SEM, * indicates p value < 0.05, ** indicates p value < 0.01, *** indicates p value < 0.001 (one-way ANOVA followed by Holm-Šídák's multiple comparisons test). h, hour.

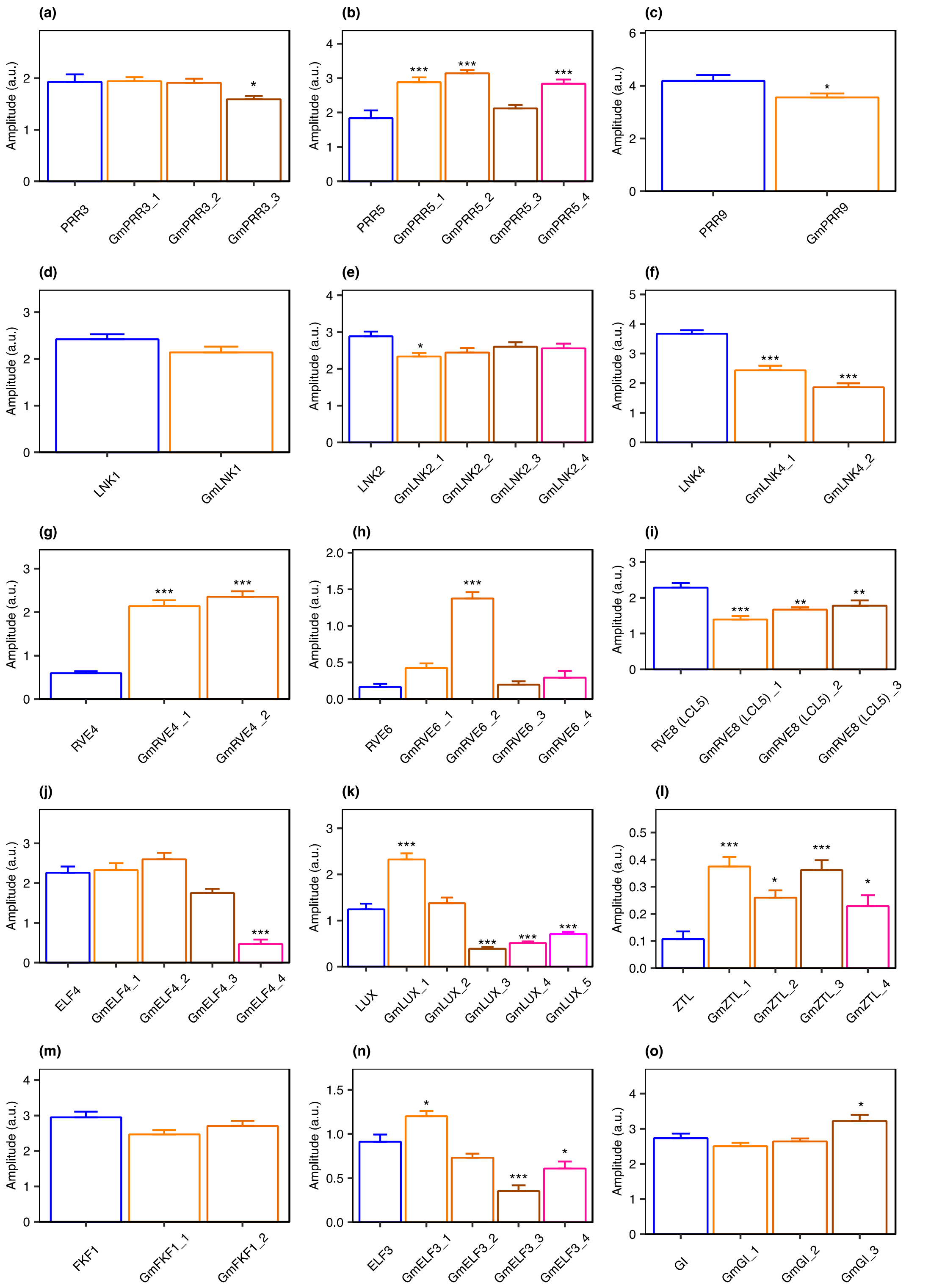

Figure S8. Amplitude of *Arabidopsis* clock genes and their homologous genes in soybean. For bar graph, data were presented as mean + SEM, * indicates p value < 0.05, ** indicates p value < 0.01, *** indicates p value < 0.001 (one-way ANOVA followed by Holm-Šídák's multiple comparisons test). a.u., arbitrary unit.

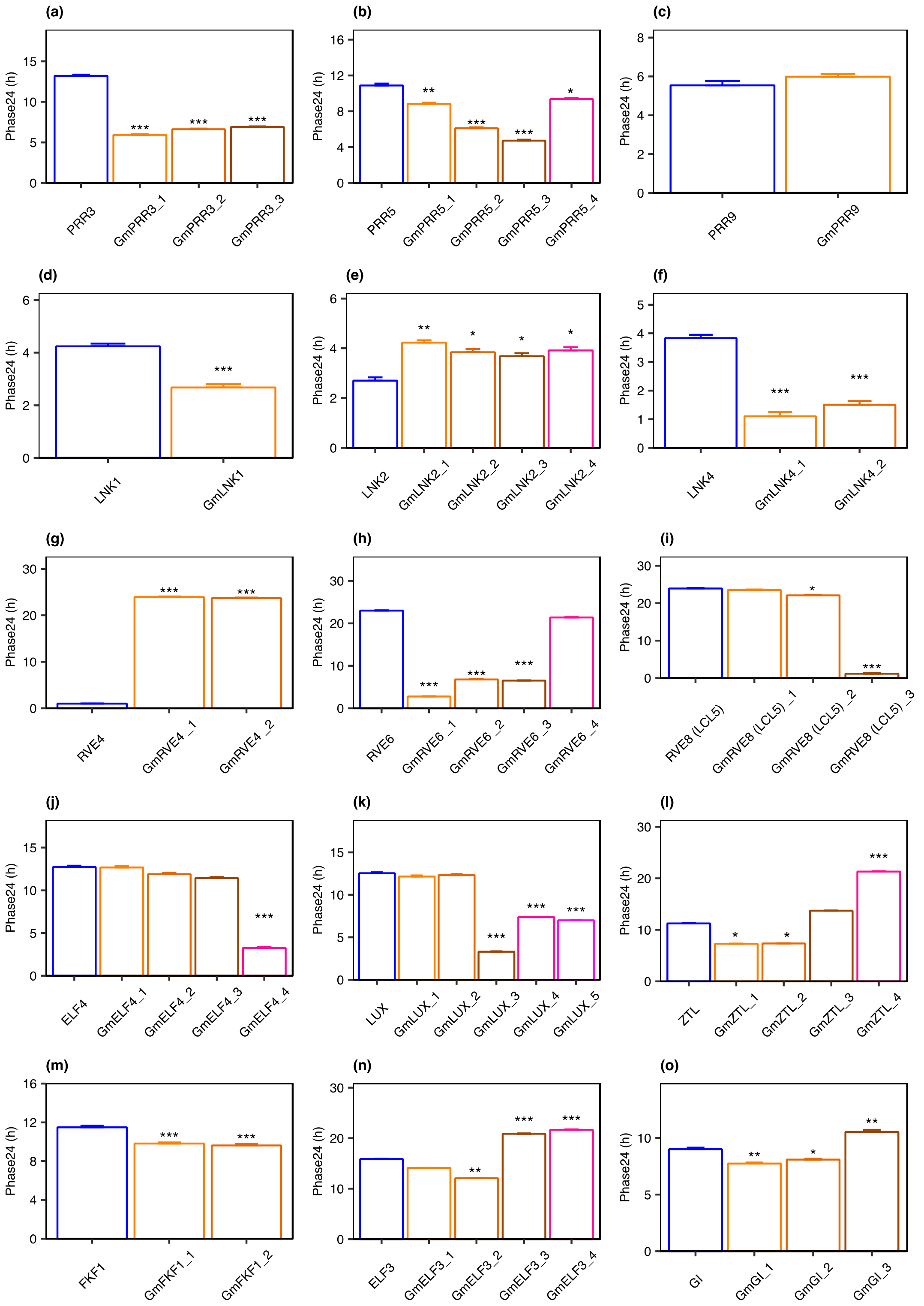

Figure S9. Phase24 of *Arabidopsis* clock genes and their homologous genes in soybean. For bar graph, data were presented as mean + SEM, * indicates p value < 0.05, ** indicates p value < 0.01, *** indicates p value < 0.001 (one-way ANOVA followed by Holm-Šídák's multiple comparisons test). h, hour.

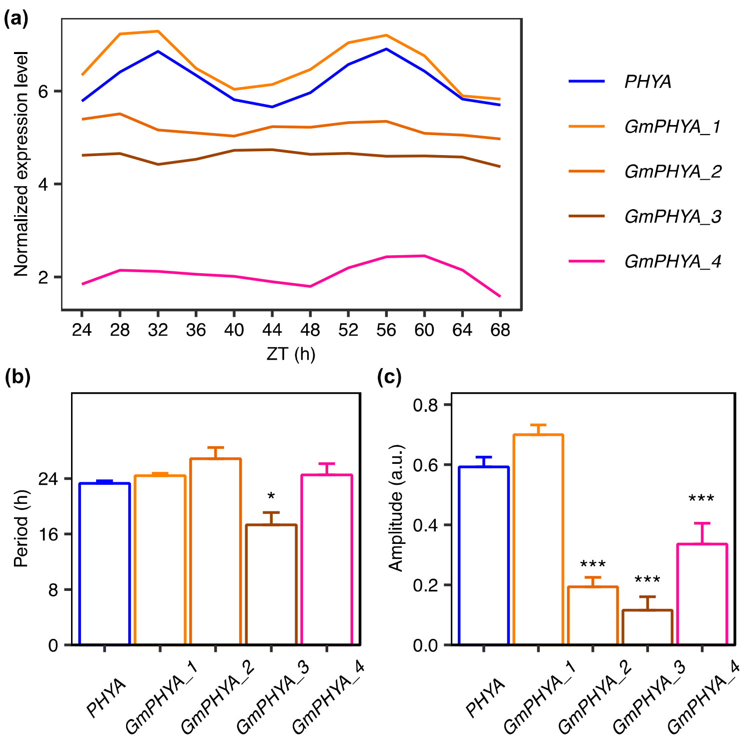

Figure S10. Comparison of *Arabidopsis* photoreceptor *PHYA* and its homologs in soybean. (**a**) Expression profiles of *Arabidopsis* gene *PHYA* and its homologous genes (From Number 1 to 4) in soybean; (**b**) Period of *Arabidopsis* gene *PHYA* and its homologous genes *GmPHYA* in soybean; **(c)** Amplitude of *Arabidopsis* gene *PHYA* and its homologous genes *GmPHYA* in soybean. For bar graph, data were presented as mean + SEM, * indicates p value < 0.05, ** indicates p value < 0.01, *** indicates p value < 0.001 (one-way ANOVA followed by Holm-Šídák's multiple comparisons test). h, hour.

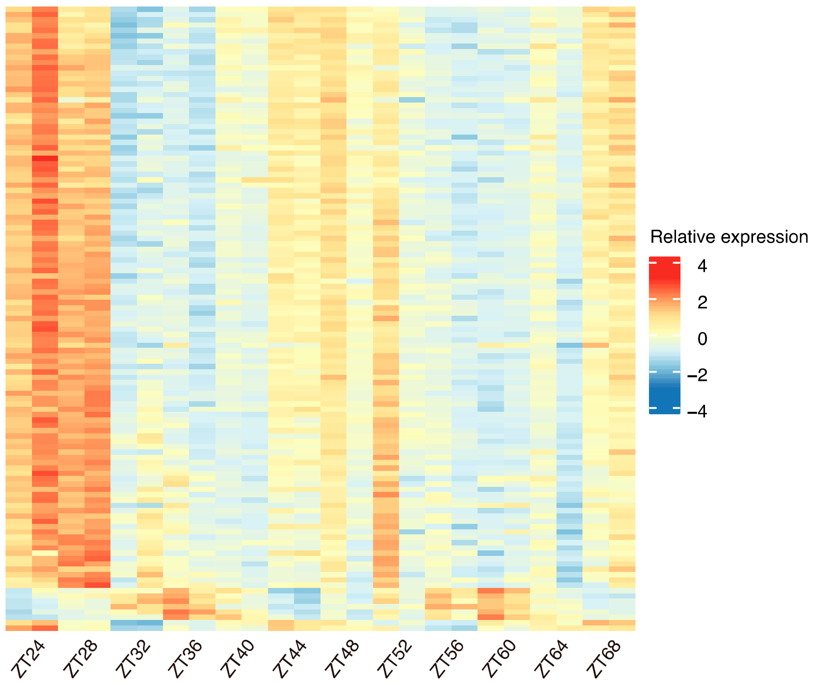

Figure S11. Heatmap showing expression of 117 circadian rhythmic genes associated with the enriched GO term “cytosolic large ribosomal subunit” in *Arabidopsis*. Genes are sorted by their phases in ascending order.

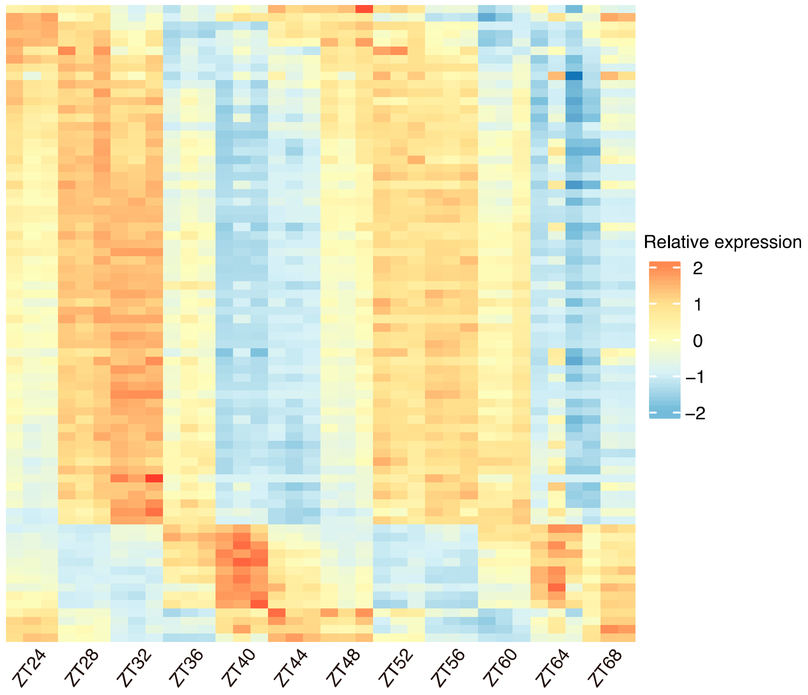

Figure S12. Heatmap showing expression of 76 circadian rhythmic genes associated with the enriched GO term “photosystem” in soybean. Genes are sorted by their phases in ascending order.

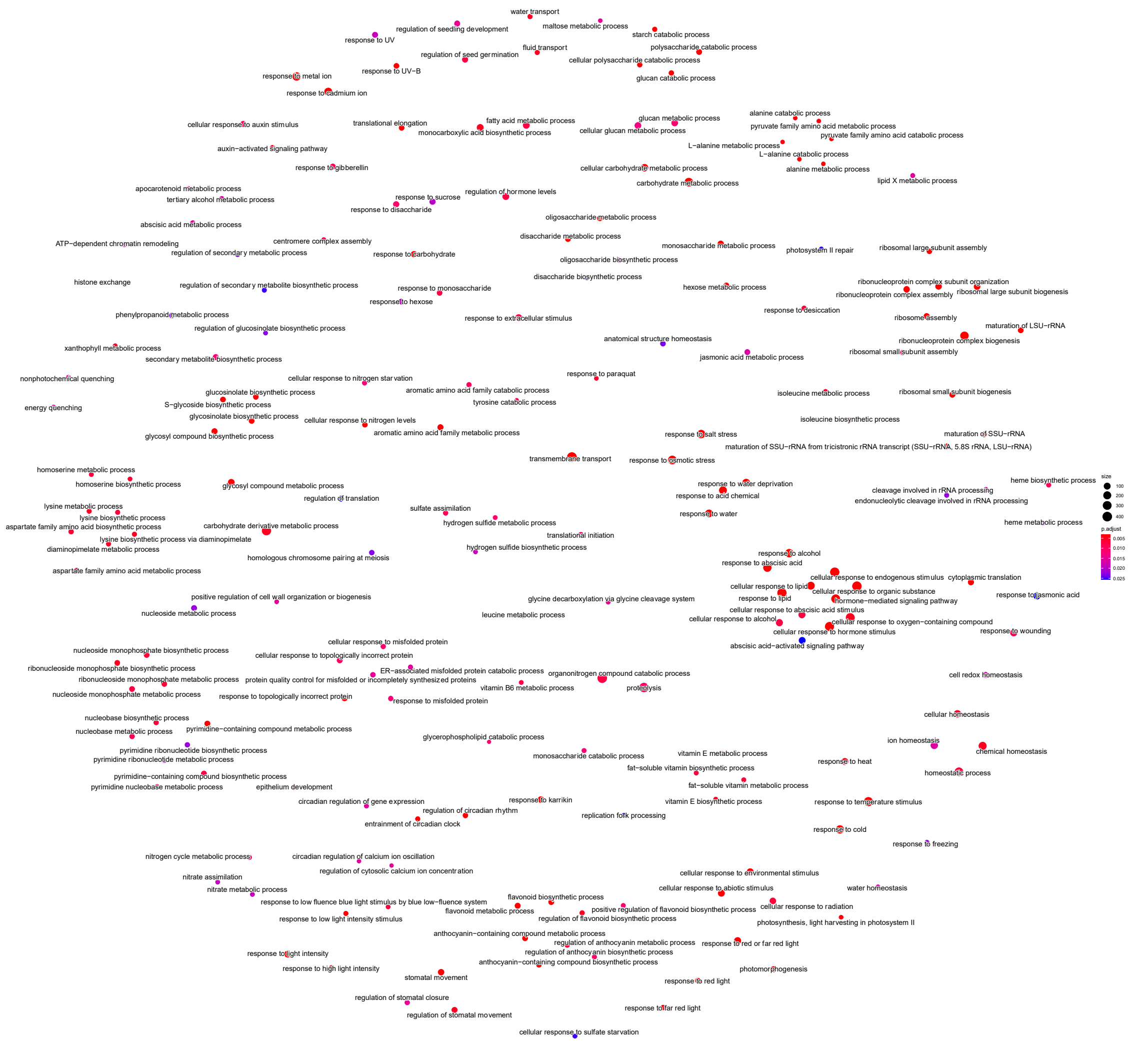

Figure S13. Relationship map of BP terms from 387 GO terms specifically enriched in *Arabidopsis* from the Venn diagram in **Fig. 8a** showing the overlapped GO terms between *Arabidopsis* and soybean derived by GSEA with FDR ≤ 0.05 as cutoff. *p.adjust* indicates FDR.

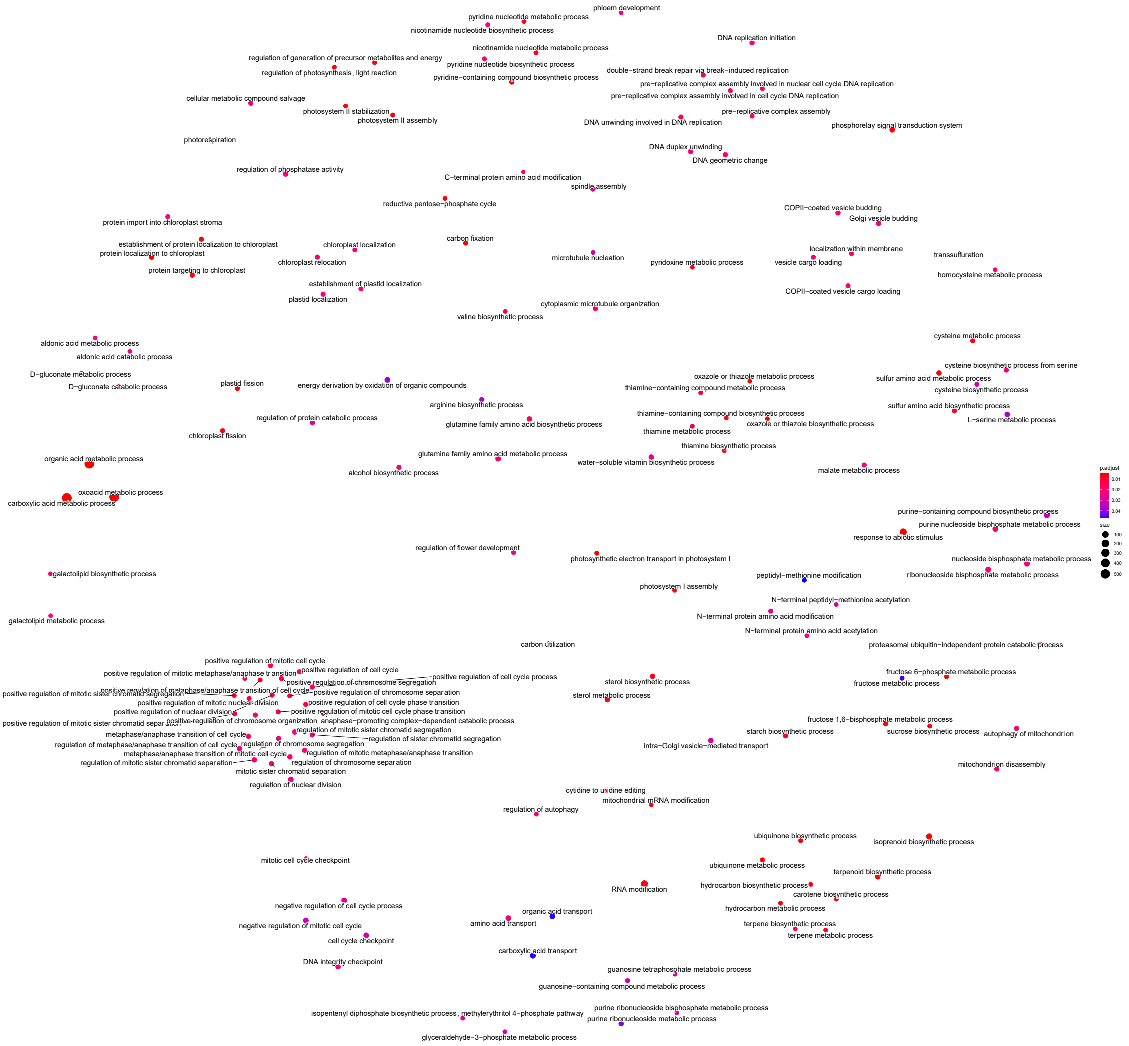

Figure S14. Relationship map of BP terms from 260 GO terms specifically enriched in soybean from the Venn diagram in **Fig. 8a** showing the overlapped GO terms between *Arabidopsis* and soybean derived by GSEA with FDR ≤ 0.05 as cutoff. *p.adjust* indicates FDR.

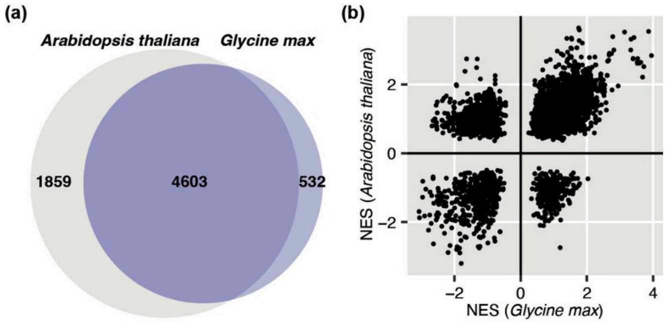

Figure S15. (**a**) Venn diagram showing overlapped GO terms between *Arabidopsis* and soybean derived by GSEA without *p* value cutoff; (**b**) Comparison of the NESs of the common 4,603 GO terms from (**a**) between *Arabidopsis* and soybean.
